# Effects of NGF Addition on Llama (*Lama glama*) Sperm Traits After Cooling

**DOI:** 10.3389/fvets.2020.610597

**Published:** 2021-01-05

**Authors:** Luciana M. Sari, Renato Zampini, Francisco Gonzalez del Pino, Martin E. Argañaraz, Marcelo H. Ratto, Silvana A. Apichela

**Affiliations:** ^1^Facultad de Bioquímica, Química y Farmacia, Instituto Superior de Investigaciones Biológicas (INSIBIO), Consejo Nacional de Investigaciones Científicas y Técnicas (CONICET), Instituto de Biología “Dr. Francisco D. Barbieri” Universidad Nacional de Tucumán (UNT), San Miguel de Tucumán, Argentina; ^2^Cátedra de Biología Celular y Molecular, Facultad de Bioquímica, Química y Farmacia, Universidad Nacional de Tucumán, San Miguel de Tucumán, Argentina; ^3^Cátedra de Zootecnia General I, Facultad de Agronomía y Zootecnia, Universidad Nacional de Tucumán, San Miguel de Tucumán, Argentina; ^4^Facultad de Ciencias Veterinarias, Instituto de Ciencia Animal, Universidad Austral de Chile, Valdivia, Chile

**Keywords:** β-nerve growth factor, seminal plasma, llama (*Lama glama*), sperm, cryopreservation, cooling

## Abstract

To provide new insights into the mechanisms through which seminal plasma proteins can protect sperm from damage caused during refrigeration, we evaluate the possibility that β-NGF can contribute to the improvement of sperm quality after cooling. First, β-NGF was detected in refrigerated sperm and compared with unrefrigerated sperm by western blotting of the proteins adsorbed by sperm, showing that native β-NGF is still present even 24 h after cooling only as an active form. Then, the effect of exogenous β-NGF on sperm quality after cooling was evaluated. A total of 12 ejaculates from male llamas (three ejaculates per male), were obtained by electro-ejaculation, diluted 4:1 with buffer Hepes-balanced salt solution and centrifuged at 800 × g for 8 min to remove the seminal plasma. Sperm were suspended in Tris-citrate-fructose-egg yolk diluent for a final concentration of 30 ×10^6^/ml and cooled at 5°C for 24 h. After refrigeration, the extended sperm were equilibrated for 5 min at 37°C and divided into the following subgroups: sperm samples without treatment (control) and sperm samples supplemented with exogenous human β-NGF (10, 100, and 500 ng/ml). At 5, 30, and 60 min of incubation sperm were evaluated for sperm viability (using eosin/nigrosin stain), sperm motility and vigor (observed under light microscopy), and mitochondrial activity (using the JC-1 fluorescent marker). Vigor data were analyzed with the nonparametric Kruskal-Wallis test. The rest of the variables were analyzed with a mixed models approach. Mean comparisons were performed using Fisher's LSD test with a confidence level of 95%. A principal components analysis was performed to analyze the relationships between variables. Treatment of 24 h cooled sperm with 10 or 100 ng/ml of human β-NGF increased the percentage of total motility and vigor (*p* < 0.05). Besides, an incubation time of 60 min would be adequate to improve sperm quality, since all variables are positively related. The significant improvement observed in the motility and vigor of post-refrigerated sperm suggests that supplementation with exogenous β-NGF may be profitable for the improvement of cooled llama sperm.

## Introduction

Artificial insemination (AI) is an important technique to ensure rapid genetic progress. To date, AI efficiency in domestic South American camelids is low (1, 2). Technical aspects of semen collection, dilution, and cryopreservation have been the main limitations for the development and use of AI in these species (1). Different protocols for sperm cooling and freezing, as well as semen extenders are currently under investigation. To date, higher pregnancy rates have been achieved using cooled sperm (3, 4) than with frozen-thawed semen (1, 5, 6).

A protocol for AI or cryopreservation of camelid semen should be designed taking into account the striking particularities of semen composition and spermatozoa. Camelid semen is highly viscous and form a threat when is pipetted (7). Unlike other domestic ruminants, the sperm movement is mainly oscillatory (8, 9), and the relationship between this characteristic and fertility has not been established yet. Seminal plasma composition seems also to be particular. Camelids account for a higher concentration of β-NGF when compared with other mammals (10). This factor is responsible for inducing ovulation in camelid females once it is deposited in the genital tract during copulation (11). In a previous study, we provided some evidence that seminal β-NGF may have roles in the regulation of llama sperm physiology (12).

When ejaculated and epididymal spermatozoa were analyzed, we found that β-NGF was present only in ejaculated sperm, indicating that this factor is provided by the seminal plasma during ejaculation (12). The presence of the main receptor of β-NGF, TrKA, in the sperm middle portion led us to propose that β-NGF might have an impact on sperm traits such as motility. Several studies performed in other species reported that the addition of β-NGF affects sperm traits. Treatment of frozen-thawed bull sperm cells with 40–80 ng/ml β-NGF increased both leptin secretion and sperm membrane integrity (13). In human sperm, addition of several concentrations of β-NGF (1–10 μM) for 30 min increased in a dose-dependent manner the sperm motility (14); and supplementation of freezing extender with β-NGF at 0.5 ng/ml improved sperm viability and motility and decreased DNA fragmentation after thawing (15, 16). The *in vitro* incubation of rabbit sperm with exogenous human β-NGF (100 ng/ml) maintained a high motility rate and track speed (17). Consistently, Bezerra et al. (18) reported a positive association between the β-NGF protein expression in seminal plasma and rabbit sperm motility.

To judge from the reports mentioned, β-NGF may be an optimal candidate protein to enhance sperm quality in camelids. Thus, the main objective of the current study was to evaluate the effect of exogenous human β-NGF on the semen quality of cooled llama sperm.

## Methods and Materials

### Animals and Location

Four male llamas ≥4 years old were used. The study was carried out at the Experimental Center for Camelid Studies of the National University of Tucuman. The Experimental Center is situated 420 meters above sea level (masl), latitude 26°88′32″ S and longitude 65°38′43″ W. Animals were kept out at pasture in pens and supplemented with bales of alfalfa and water was provided *ad libitum*. All males were shorn during November.

### Semen Collection

Semen was obtained during the winter-spring of 2019 at regular intervals of 14 days. Collections were performed under general anesthesia with 0.2 mg/kg of xylazine IV (Xilazina, Richmond, Argentina) and 1.5 mg/kg of ketamine IV (Ketamina, Holliday, Argentina). All procedures were in line with the UNT 002/18 Protocol approved by the Committee for the Care and Use of Laboratory Animals (CICUAL) from the Universidad Nacional de Tucumán, Argentina.

For electroejaculation, a stimulator similar to an Electrojac V (Sistel, Argentina) with a rectal probe with three linear electrodes was used. The probe was lubricated, gently inserted into the rectum, and oriented so that the electrodes were positioned ventrally to the prostate. According to Zampini et al. (19), the device was used in automatic mode, applying stimulation cycles of 2 s with 2 s intervals between stimuli. Voltage was increased one volt every five cycles (starting with 2 V) until erection occurred (~8 V). Then, voltage was increased with 1 V increments every ten cycles until ejaculation.

Semen was collected in 50 ml Falcon tubes and immediately placed in a 37°C water bath.

Immediately after collection, semen samples were diluted with HBSS (25 mM Hepes, 130 mM NaCl, 5 mM KCl, 0.36 mM NaH_2_PO_4_, 0.49 mM MgCl_2_, and 2.4 mM CaCl_2_; pH 7.4, 290 mOsm/kg) at 37°C and centrifuged at 800 × g for 10 min at room temperature to remove the seminal plasma.

### Sperm Cooling

Washed sperm were kept at 37°C and suspended in 200–300 μl of HBSS, and concentration, sperm viability, motility, and vigor were assessed as described below. Then, Tris citric acid fructose-egg yolk extender (TCF-EY: 250 mM Tris, 80 mM citric acid, 60 mM fructose, 20% egg yolk, 0.5% Equex, 80,000 IU Penicillin G sodium, and 0.1% Streptomycin sulfate) was added to obtain a final concentration of 30–40 × 10^6^ spermatozoa/ml. Samples were subsequently placed in a 37°C water bath and then placed in a refrigerator. Temperature was monitored until reaching 5°C in ± 2.5 h (cooling rate 0.2°C/min). Samples were then maintained at 5°C for 24 h. After this period, the cooled sperm cells were warmed up to 37°C in a water bath.

### Experiment 1: Detection of β-NGF in Cooled Llama Spermatozoa

#### Extraction of Sperm Adsorbed Protein

Sperm-adsorbed proteins (SAP) were obtained from fresh-washed sperm and 24 h cooled sperm, as previously described by Zampini et al. (20). Briefly, pools of six semen samples (*n* = 3) for each experimental group were diluted 5-fold and washed three times at 800 × g for 10 min with HBSS. The sperm pellets were suspended in HBSS with 1X protease inhibitor cocktail (Sigma). An equal volume of 1 M KCl in HBSS was added, and sperm proteins were extracted by gentle mixing on a rotary shaker for 1 h at 4°C. Spermatozoa were then removed by centrifugation at 6,000 × g for 10 min, and discarded. Extracts were clarified by filtration (0.2-μm cellulose acetate). A series of centrifugations in 3 kDa cellulose filters (Amicon, Lexington, MA) were performed at 9,000 × g at 4°C, re-diluting in HBSS every centrifugation to reduce the salts and to concentrate the sample. Protein content was determined using a BCA protein assay kit (Thermo Fisher Scientific, Rockford, IL) with a bovine serum albumin standard. Aliquots were stored at −70°C until needed.

#### Western Blotting

Western blotting was performed according to Sari et al. (12). Ten micrograms of total sperm-adsorbed proteins of fresh and cooled sperm (*n* = 3) obtained as indicated before, were subjected to 15% SDS-PAGE along with 100 ng of recombinant human β-NGF (R&D Systems, Minnesota, USA) in a separate well. Gels were run in a PROTEAN II xi Cell (Bio-Rad, Hercules, CA) at 150 V for 1.5 h at room temperature. Then, proteins were transferred onto PVDF membranes (Immobilon-P, Merck, Darmstadt, Alemania). Semi-dry electroblotting was carried out on a Trans-Blot SD semi-dry transfer cell (Bio-Rad; Richmond, CA) and run at 20 V for 30 min. The membranes were blocked in 5% BSA in HBSS-T (130 mM NaCl, 5 mM KCl, 1.36 mM Na_2_HPO_4_, 2.4 mM CaCl_2_, 25 mM HEPES, 0.49 mM MgCl_2_, 0.1% Tween 20) for 30 min at 37°C and then overnight at 4°C. Afterward, membranes were incubated with polyclonal antibodies against β-NGF (dilution 1:2,000, sc-548) (Santa Cruz Biotechnology) for 2 h at 37°C, followed by incubation with the secondary antibody for 1 h at 37°C (1:1,000) (biotinylated anti-rabbit IgG antibody, B8895, Sigma). Finally, membranes were incubated for 30 min with alkaline phosphatase-linked ExtrAvidin (1:5,000, Sigma), and protein bands were visualized using the SigmaFast substrate (BCIP/NBT, Sigma). Membrane digital images were obtained with a Pentax Optio M90 digital camera. Molecular weight marker proteins (Page Ruler Unstained Broad Range Protein Ladder, Thermo Fisher Scientific, Rockford, IL) were also run and transferred to determine the molecular weights of the labeled bands. The primary antibody omitted from the staining reaction was used as control.

### Experiment 2: Effect of β-NGF Addition on Llama Sperm Traits

#### Sperm Preparation Samples

Twelve ejaculates were collected from 4 llamas (*n* = 4, *r* = 3). After 24 h of cooling, sperm samples were warmed at 37°C. Minimum viability of 55% of live sperm and absence of agglutinated sperm were verified before beginning the experiment. Then, each sperm sample was divided into four treatment groups: without addition of exogenous β-NGF (control group) and with addition of 10, 100, or 500 ng/ml of human recombinant β-NGF (R&D Systems, Minnesota, USA). Treatment groups were incubated at 37°C for 60 min. At 5, 30, and 60 min of incubation, aliquots were taken to assess the viability, motility, and vigor. Sperm mitochondrial function was evaluated after 60 min of β-NGF addition.

#### Assessment of Viability

The vital eosin-nigrosin staining was used for assessing sperm viability. Briefly, 10 μl of the sperm suspension were placed on a slide with an equal volume of dye (2 eosin: 3 nigrosin), mixed, and then smeared. Once dried, slides were examined under a bright-field microscope (Numak, model Zenith DO-1L, Buenos Aires, Argentina) at 400X. Live spermatozoa appeared unstained, whereas dead spermatozoa with disrupted membranes appeared stained in red. Two hundred spermatozoa where examined on each slide (*r* = 2), averaged, and expressed as percentage of live spermatozoa.

#### Assessment of Motility and Vigor

To evaluated sperm motility and vigor, 7 μl of the sperm suspension were placed in a slide under a heating stage, coved with a warmed coverslip (18 × 18 mm), and observed under a bright-field microscope (Numak, model Zenith DO-1L, Buenos Aires, Argentina) at 400X. Sperm motility was expressed as the percentage of total motility i.e., progressive plus oscillatory.

For assessment of vigor, sperm movement was scored from 0 to 5 following Table 1 criteria, based on the number of sperm with movement, the frequency of oscillatory movement of the sperm, and the presence of progressive movement.

**Table 1:** Sperm vigor score.

**Score**	**Description**
0	Sperm have no movement.
1	Total motility is poor. Very few sperm (about 10%) have weak movements.
2	Sperm have weak oscillatory movement. Progressive motility is not observed.
3	Sperm have mainly oscillatory movement, with slow beat frequency, and low progressive movement. Less than 40% of cells are motile.
4	Sperm have vigorous oscillatory movement, with fast beat frequency. About 40–50% of cells are motile.
5	Sperm have very fast oscillatory or progressive movement; 60% of cells are motile.

#### Assessment of Mitochondrial Activity

Mitochondrial membrane potential (MMP) was evaluated by using JC-1 dye (CAS 3520-43-2, Santa Cruz Biotechnology, USA). The JC-1 probe accumulates in the mitochondria as a fluorescent green monomer in inactive mitochondria, and when mitochondria exhibit high membrane potential (active mitochondria) the monomers form aggregates that shift to fluorescent orange.

For MMP assessment, 150 μl of each treatment group (30 × 10^6^ sperm/ml) of were incubated with β-NGF as previously described. After 15 min of incubation, 3 μl of JC-1 (1.53 mM) was added and then incubated for a further 45 min. Then, the sperm were washed in HBSS and suspended in 150 μl of HBSS. Fluorescence was determined in a fluorometer (Perkin Elmer LS55) using 590-nm excitation/525-nm emission for the J-aggregate of JC1, and 530-nm excitation/490-nm emission for the J-monomer. MMP was calculated as J-aggregate/J-monomer ratio.

#### Statistical Analysis

The effects of β-NGF and time on sperm viability, motility, and mitochondrial activity were determined using a linear mixed-effects model (21) with the R program through the graphical interface developed for Infostat (22). In the model, the llama was used as a random effect to account for the documented inter-llama variability (23). *Q-Q* plots were used to check for departures from the normal distribution. Differences between media treatments were compared with Fisher's least significant difference (LSD) test. The same effects on vigor were evaluated using the Kruskal-Wallis test. Data are presented as mean ± standard error (SE), except for vigor, which was expressed as the median, and the minimum significance level was *p* < 0.05.

A multivariate method of statistical analysis, principal component analysis (PCA), was applied in addition to the conventional tests used for univariate analysis. The PCA offers the advantage of allowing rapid visualization of the relevant information on a single scatter plot graph including all the sperm traits assayed and β-NGF concentrations at the same time. Separate models were used for 5, 30, and 60 min data. The results of the PCA were displayed in a two-dimensional graph with projections of the sperm traits variables (vectors) in the factor space in which the horizontal and vertical axis represents the principal factors. The angles between the vectors indicate the correlations between the variables, where the length of the vectors indicates the contribution of the variables to the total variance of the data matrix (24).

## Results

### Detection of β-NGF in Cooled Llama Spermatozoa

**Figure 1:**
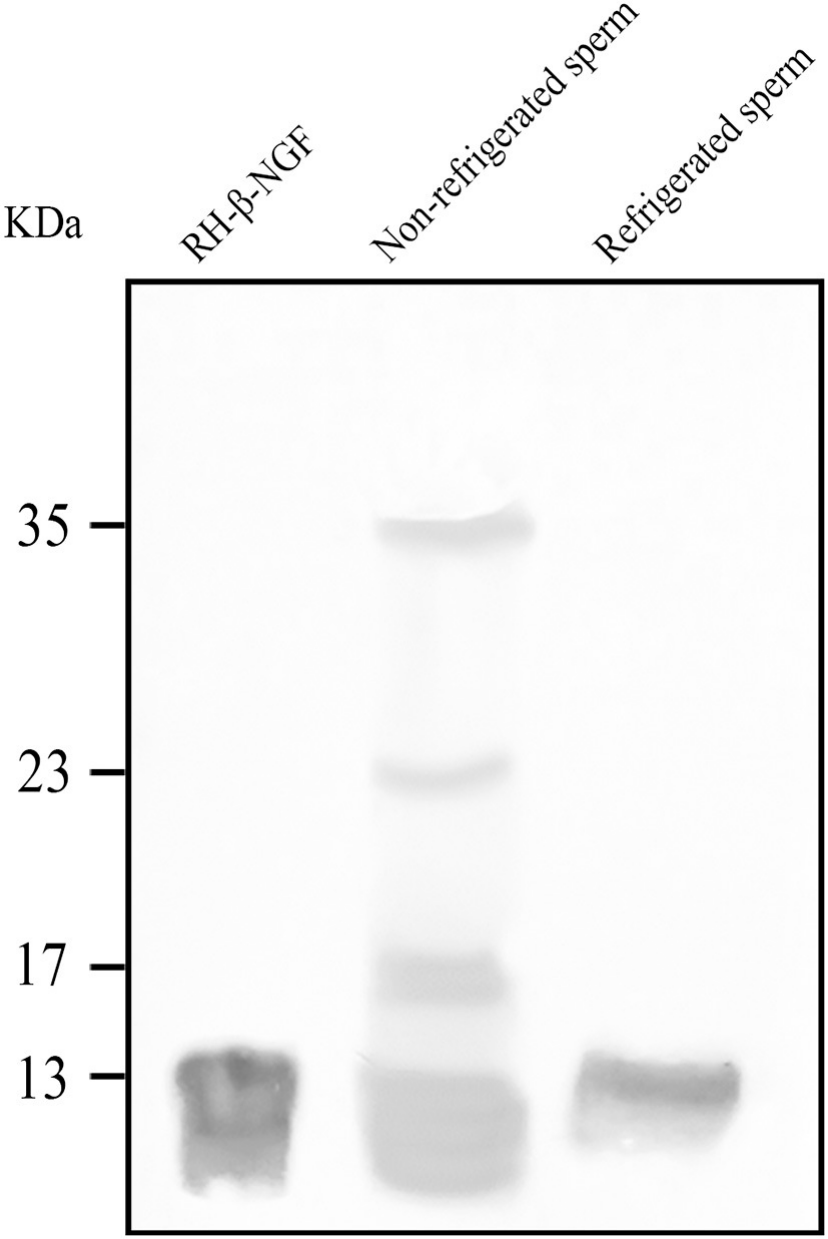
Western blotting of β-NGF protein in llama sperm-adsorbed protein (SAP) of both, fresh and 24 h refrigerated spermatozoa. At positive control, 100 ng of recombinant human β-NGF (RH- β-NGF) was run.

Western blotting revealed the presence of the β-NGF protein in the sperm adsorbed proteins of both, fresh and 24-h refrigerated spermatozoa (Figure 1). SAP of unrefrigerated samples presented four β-NGF species of 13, 17, 23, and 35 kDa, whereas refrigerated samples presented a unique protein band of 13 kDa.

### Effect of β-NGF on Llama Sperm Traits

The effect of different concentrations of β-NGF on sperm viability was evaluated at three incubation times as shown in Table 2. At 5 min of incubation, no differences (*p* > 0.05) were observed for viability, whereas at 30 min of incubation, viability in control samples resulted higher than in 10 ng/ml β-NGF group (*p* < 0.05). Conversely, at 60 min after incubation, sperm samples with 10 ng/ml β-NGF added presented a higher percentage of live spermatozoa than those without β-NGF (*p* < 0.05).

**Table 2:** Percentage of llama sperm viability.

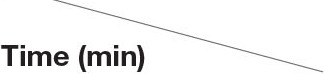 β-NGF (ng/ml)	**0**	**10**	**100**	**500**
5	74.70 ± 2.53^a^	74.26 ± 2.50^a, b^	71.90 ± 2.50^a, b, c^	71.17 ± 2.50^a, b, c, d^
30	71.90 ± 2.50^a, b, c^	67.81 ± 2.50^d, e, f^	69.81 ± 2.50^c, d, e, f^	70.72 ± 2.50^b, c, d^
60	66.44 ± 2.50^f^	70.26 ± 2.50^c, d, e^	68.17 ± 2.50^d, e, f^	66.90 ± 2.50^e, f^

*Values are mean ± standard error for the concentration-time factor; n = 12 for each treatment*.

a, b, c, d, e, f *Means with the same letter are not significantly different from each other (p > 0.05 Linear Mixed-Effects Models followed by LSD Fisher test)*.

**Figure 2:**
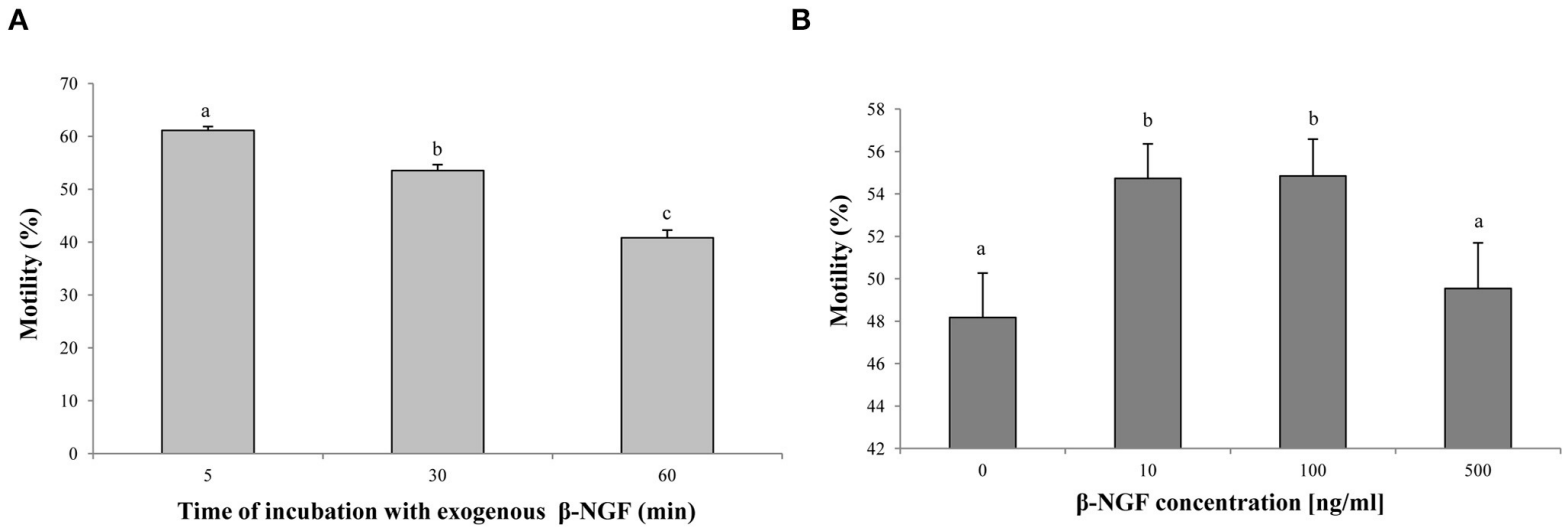
Effect of β-NGF on llama sperm motility after cooling. **(A)** Incubation time-effect (*p* < 0.05, *n* = 48 for each time point). **(B)** Concentration effect (*p* < 0.05, *n* = 36 for each treatment). Results are expressed as mean ± SE. ^a, b^Different letters indicate significant differences.

Regarding sperm motility, the interaction between β-NGF concentration and time was not significant (interaction *p* = 0.4940) indicating that the effect of β-NGF addition on sperm motility is independent of the incubation time. Therefore, they were analyzed separately. Sperm motility parameters decreased over incubation time (*p* < 0.05) (Figure 2A). Media containing 10 and 100 ng/ml of β-NGF were superior showed a marked increase in total motility to the extenders containing 0 and 500 ng/ml of β-NGF (*p* < 0.05) (Figure 2B).

As shown in Table 3, the addition of 100 ng/ml of β-NGF increased significantly vigor after incubation for 30 or 60 min at 37°C when compared with the control group without β-NGF (*p* < 0.05).

**Table 3:** Percentage of llama sperm vigor (scale 0–5) after incubation with exogenous β-NGF.

**β-NGF (ng/ml)**	**Time (min)**		
	**5**	**30**	**60**
0	4 ± 0.30^a^	3 ± 0.23^a, b^	2 ± 0.16^a^
10	4 ± 0.28^a^	4 ±0.15^c^	3^a, b^
100	4 ± 0.16^a^	4 ± 0.12^c^	3 ± 0.19^b^
500	4 ± 0.21^a^	3 ± 0.14^b^	3 ± 0.21^a, b^

*Data are expressed as median ± standard error*.

a, b, c *Within columns, different letters between rows indicate significant differences (p < 0.05, n = 11 per group)*.

**Figure 3:**
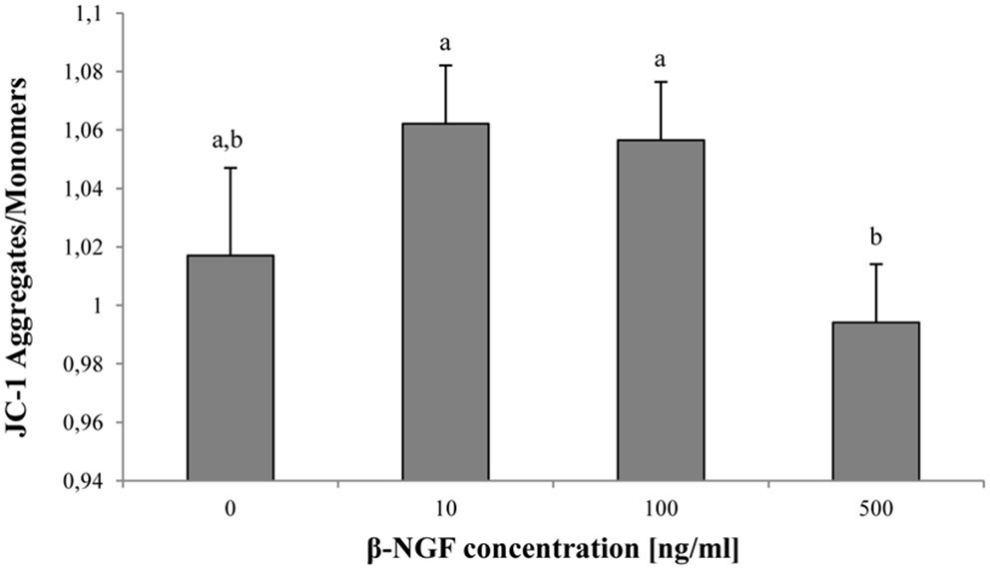
Mitochondrial activity at 60 min of incubation with β-NGF. Bars represent the mean of JC-1 aggregate/monomer ratio ± SE, *n* = 12 for each treatment. ^a, b^Different letters indicate significant differences (*p* < 0.05).

Regarding mitochondrial activity, a decrease of MMP was observed when 500 ng/ml of β-NGF were added, compared with 10 or 100 ng/ml of β-NGF (*p* < 0.05). Nevertheless, no differences with the control were found (Figure 3).

**Figure 4:**
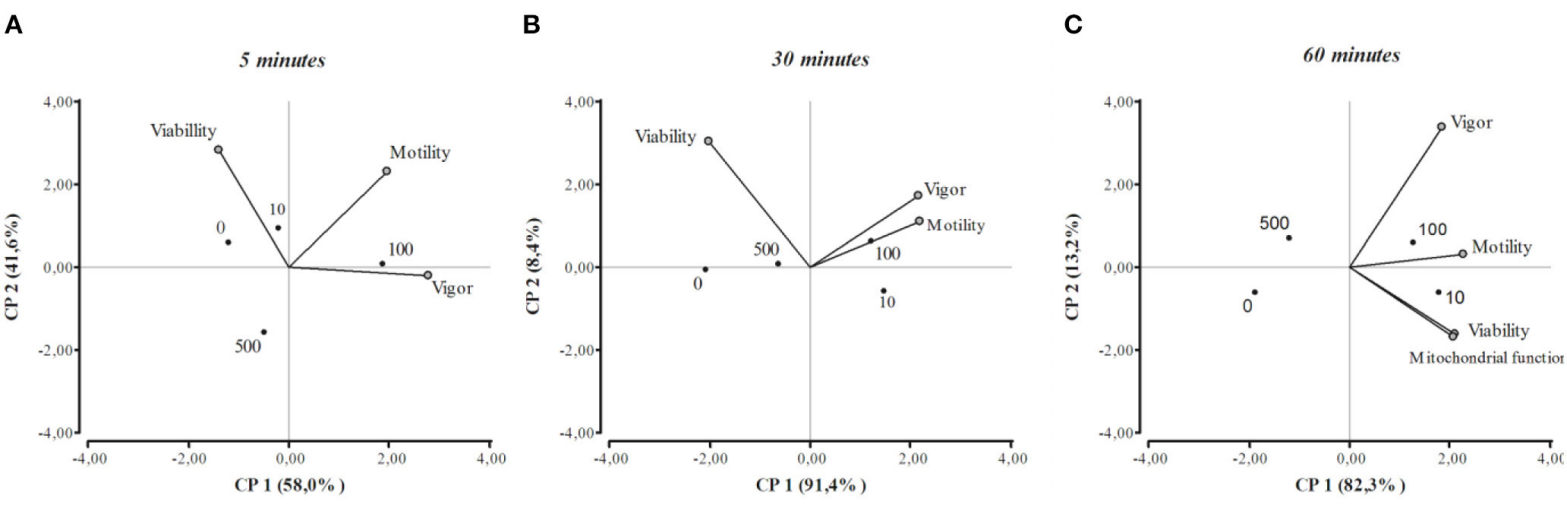
Principal component analysis biplot at 5, 30, and 60 min of incubation with β-NGF **(A–C)**, showing the first two principal components (PC1 and PC2). Vectors indicate the relations between viability, motility, sperm vigor, and mitochondrial function. Each vector points in the direction of the increase in value. The dots represent the mean value for each concentration of β-NGF (0, 10, 100, 500 ng/ml).

The results of the PCA are shown in a two-dimensional graph (Figure 4). The length of the vectors represents the contribution of the variables to the total variance of each factor. When two variables were close to each other they were positively correlated. Otherwise, if they were orthogonal there was no correlation, if the variables were on the opposite side of the graph, they were negatively correlated. After 5 or 30 min from β-NGF addition, the motility and vigor of the sperm were close to each other in the right quadrant, indicating a positive interrelation between them, this association being closer after 30 min of incubation. It can also be appreciated that the 100 ng/ml dose of β-NGF induced the highest values of motility and vigor at these time points. Regarding sperm viability, the variable correlated negatively with vigor and motility at 5 and 30 min of β-NGF addition. At 60 min after sperm incubation with β-NGF, it is observed that all variables are positively correlated, and the values of these variables were higher when 10 or 100 ng/ml of β-NGF was added.

## Discussion

As described in the method section, before refrigeration the native source of β-NGF, the seminal plasma, was removed, and spermatozoa were extended in an appropriated diluent. Western blotting of sperm-adsorbed proteins revealed that after 24 h of cooling llama β-NGF was still adsorbed by spermatozoa, indicating a strong time-sustained affinity. However, there seems to be some degree of processing of the protein into an active form, since the pro-β-NGF isoforms are absent. Further studies would be necessary to define whether this factor remains functional. This is the first report about the presence of β-NGF adsorbed to cooled sperm conditions.

To assess the effect of β-NGF supplementation, exogenous human β-NGF was added. Because the llama sperm TrKA-receptor was localized in the middle piece (12), it was hypothesized that β-NGF would directly affect the sperm motility improvement. To judge from the conditions in the present study, the addition of β-NGF improved the quality of cooled spermatozoa.

Motility of cooled llama sperm increased with the addition of 10 and 100 ng/ml of exogenous β-NGF, and this positive effect was independent of the incubation time. Several reports in other mammals indicate that β-NGF can promote sperm motility parameters of ejaculated sperm and in a time- and dose-dependent manner—human (14, 25); bull (13); rabbit (17). On the other hand, 100 ng/ml of β-NGF was the optimal dose to improve sperm motility in rabbits (17), and 10 ng/ml of β-NGF or less for human sperm (14, 15, 24). In South American camelids, ejaculates present mainly oscillatory sperm motility (23), and in some cases the differences between treatments can be reflected in the frequency of oscillatory movement (vigor) more than in the progressive movement. For this reason, sperm score vigor was included. As expected, β-NGF treatment enhanced sperm affected vigor, occurring consistently at 30 and 60 min of incubation with a concentration of 100 ng/ml of β-NGF.

With respect to viability, the effect of β-NGF addition was dose- and time-dependent since time is mainly responsible for sperm viability changes. β-NGF supplementation does not seem to negatively affect sperm viability in spermatozoa at any dose, although some statistically significant differences between treatments were observed. We found a positive effect at 60 min of incubation with 10 ng/ml of β-NGF. Improvement of sperm viability has been reported in other species. Bull spermatozoa presented increased sperm viability when 20, 80, or 120 ng/ml of β-NGF was added (13), whereas in rabbits 100 ng/ml of β-NGF increased sperm viability (17). This response would be mediated by β-NGF binding to the TrkA receptor (17).

As mentioned above, in the llama spermatozoa the TrKA receptor is located in the midpiece, suggesting that receptor localization is directly involved in mitochondrial function. In this study, the JC-1 dye was used due to its ability to discriminate high and low mitochondrial membrane potential (26). The addition of β-NGF (10, 100, 500 ng/ml) did not affect the MMP. Similarly, Li et al. (13) reported that increasing levels of β-NGF (from 20 to 120 ng/ml) did not affect the mitochondrial activity of bull spermatozoa. It has been proposed that a prolonged fall or increase in the MMP compared to normal levels can induce an unwanted loss of cell viability (27). Since no negative effects were observed in MMP as well as sperm viability, working concentrations of 10–500 ng/ml of β-NGF could be considered secure for the llama sperm in the conditions of the present study.

To clarify the role of β-NGF in llama sperm physiology, the relationship between sperm traits and β-NGF doses were characterized by principal component analysis. It was noticed that the relationship between viability, motility, sperm vigor, and mitochondrial function was positive only at 60 min of incubation with β-NGF, and improvement of all of these traits can be achieved with both 10 and 100 ng/ml of exogenous β-NGF. These results are encouraging for their application to artificial insemination. In this sense, it would be of interest to evaluate the sperm responsiveness to β-NGF when it is incubated for a longer time, as well as the effect on capacitation and acrosome reaction of having a better prediction of the β-NGF impact on the sperm-fertilizing capacity and then conducting artificial insemination experiments.

In conclusion, β-NGF increases sperm quality of cooled llama sperm. The addition of 10 or 100 ng/ml of human β-NGF would promote motility and vigor, while viability and mitochondrial activity are maintained. Supplementation with exogenous β-NGF may be profitable for successful refrigeration of llama sperm and assisted reproduction techniques.

## Data Availability Statement

The original contributions presented in the study are included in the article/supplementary materials, further inquiries can be directed to the corresponding author/s.

## Ethics Statement

The animal study was reviewed and approved by Committee for the Care and Use of Laboratory Animals (CICUAL) from the Universidad Nacional de Tucumán, Argentina. UNT 002/18 Protocol.

## Author Contributions

SA, MR, and LS conceived and designed the study. SA and LS performed data interpretation and wrote the draft manuscript. RZ, LS, MA, and SA collected semen samples. LS performed western blot, semen refrigeration, sperm supplementation, MMP assessment, and the statistical analysis. FG and SA performed motility and vigor assessment. RZ and MA performed vitality assessment. RZ and LS performed protein extractions. MA, MR, and RZ revised and discussed the manuscript. All authors contributed to the article and approved the submitted version.

## Conflict of Interest

The authors declare that the research was conducted in the absence of any commercial or financial relationships that could be construed as a potential conflict of interest.
